# First evidence of the Hepatitis E virus in environmental waters in Colombia

**DOI:** 10.1371/journal.pone.0177525

**Published:** 2017-05-16

**Authors:** Paula A. Baez, Maria Camila Lopez, Alejandra Duque-Jaramillo, Dioselina Pelaez, Francisco Molina, Maria-Cristina Navas

**Affiliations:** 1 Grupo de Gastrohepatologia, Facultad de Medicina, Universidad de Antioquia, Medellin, Colombia; 2 Laboratorio de Virología, Instituto Nacional de Salud, Bogotá D.C., Colombia; 3 Grupo de Investigación en Gestión y Modelación Ambiental GAIA, Universidad de Antioquia, Medellin, Colombia; Centers for Disease Control and Prevention, UNITED STATES

## Abstract

Hepatitis E virus (HEV) is one of the main causes of acute viral hepatitis of enteric transmission. HEV has been detected in environmental samples in several countries from Europe and Asia, constituting a risk factor for waterborne infection. In Colombia, HEV has been identified in samples obtained from patients as well as from swine, but no environmental studies have been carried out. To determine if HEV is present in environmental waters, samples from the main source of drinking water plant and of wastewater system of eight municipalities and two villages of Antioquia state (North West Colombia), were collected between December 2012 and April 2014. The HEV genome was detected by RT-PCR in 23.3% (7/30) of the samples from the main source of drinking water plants and in 16.7% (5/30) from sewage. Viral concentrates obtained from three positive sewage samples were used to inoculate HepG2 cell cultures that were followed for one month; however, the viral genome was not detected in any cell culture. This study demonstrates the circulation of HEV in both source of drinking water plants and wastewater in Antioquia state, Colombia. The presence of HEV in environmental waters could be a risk for waterborne transmission in this population. The findings of the present study, together with the evidence of HEV circulation in human and swine in Colombia, should be consider by public health authorities for the development of surveillance programs and the inclusion of HEV infection diagnosis in the guidelines of viral hepatitis in the country. This is the first report of HEV in environmental samples in Colombia and the second one in Latin America.

## Introduction

Hepatitis E virus (HEV) is an etiologic agent of enteric acute viral hepatitis with worldwide distribution. The World Health Organization (WHO) estimates 20 million Hepatitis E infections, over 3 million symptomatic cases and 56,600 HEV-related deaths each year [1]. Around one third of the world population has been infected with HEV [2], making it one of the main causes of acute viral hepatitis, which in most cases follows a self-limited course [3]. Mortality rate is 0.5–4%; however, the mortality in pregnant women infected with HEV genotype 1 is remarkably high as described in some countries in Asia and Africa [4,5].

HEV (*Orthohepevirus A*) belongs to the *Hepeviridae* family, genus *Orthohepevirus*. It is a non-enveloped, single-stranded, positive-sense RNA virus, with a genome of 7.2 Kb [6,7]. Four genotypes of HEV infecting humans have been described [4,7]: genotypes 1 and 2 are responsible for epidemics outbreaks, mainly in developing countries, and have been isolated from humans exclusively [8], while genotypes 3 and 4 are related to sporadic cases and have been isolated from both humans and animals in non-endemic regions [3,9].

The main transmission route for HEV is fecal-oral, mainly for consumption of contaminated water [5,10]. Indeed, the presence of HEV has been demonstrated in several studies performed in environmental water, river and wastewater samples in America, Asia and Europe [11–15].

Moreover, HEV sequences detected in surface water and waste water are found to cluster with sequences obtained from indigenous cases in patients and in infected swine and wildlife animals from the same geographical region [5]. In addition to drinking water, irrigation water can be contaminated, resulting in contamination of crops [16,17]. The HEV viral particle is very resistant and remains intact under environmental conditions, facilitating its transmission [18–20].

Zoonotic transmission due to contact with infected animals and consumption of contaminated undercooked or raw meat has been identified as an important risk factor, especially in industrialized countries [5,10]. The swine is consider the primary reservoir of HEV, but the virus has also been detected in deer, wild boar, shellfish and other bivalves [5].

HEV infection has been reported in several Latin American countries (reviewed in [7,21]), with HEV genotype 3 being the most frequent genotype in the region, identified both in humans and pigs [21–24]. Noteworthy, HEV genotype 3 was recently identified in river and sewage samples in Argentina [11]. Additionally, genotype 1 has been found in human samples from Cuba, Mexico, Uruguay and Venezuela, while genotype 2 has only been described in outbreaks in Mexico between 1986 and 1987 [21,25–28]. Genotype 4 has not been reported in Latin America.

In Colombia, serological and molecular evidence of HEV infection in patients with clinical diagnosis of viral hepatitis has been demonstrated [29,30], as well in workers from swine farms [31,32]. Besides, serological and molecular evidence of HEV infection was established in fecal and liver samples of swine [33–35].

The aim of the present study was to evaluate the circulation of HEV in environmental samples obtained from the source of drinking water plant and from wastewater system in the nine regions of Antioquia state, Northwest Colombia. This is the first report of HEV circulation in environmental samples in Colombia, and the results contribute to the epidemiology of the virus in the country and the region.

## Materials and methods

### Study design

**Fig 1:**
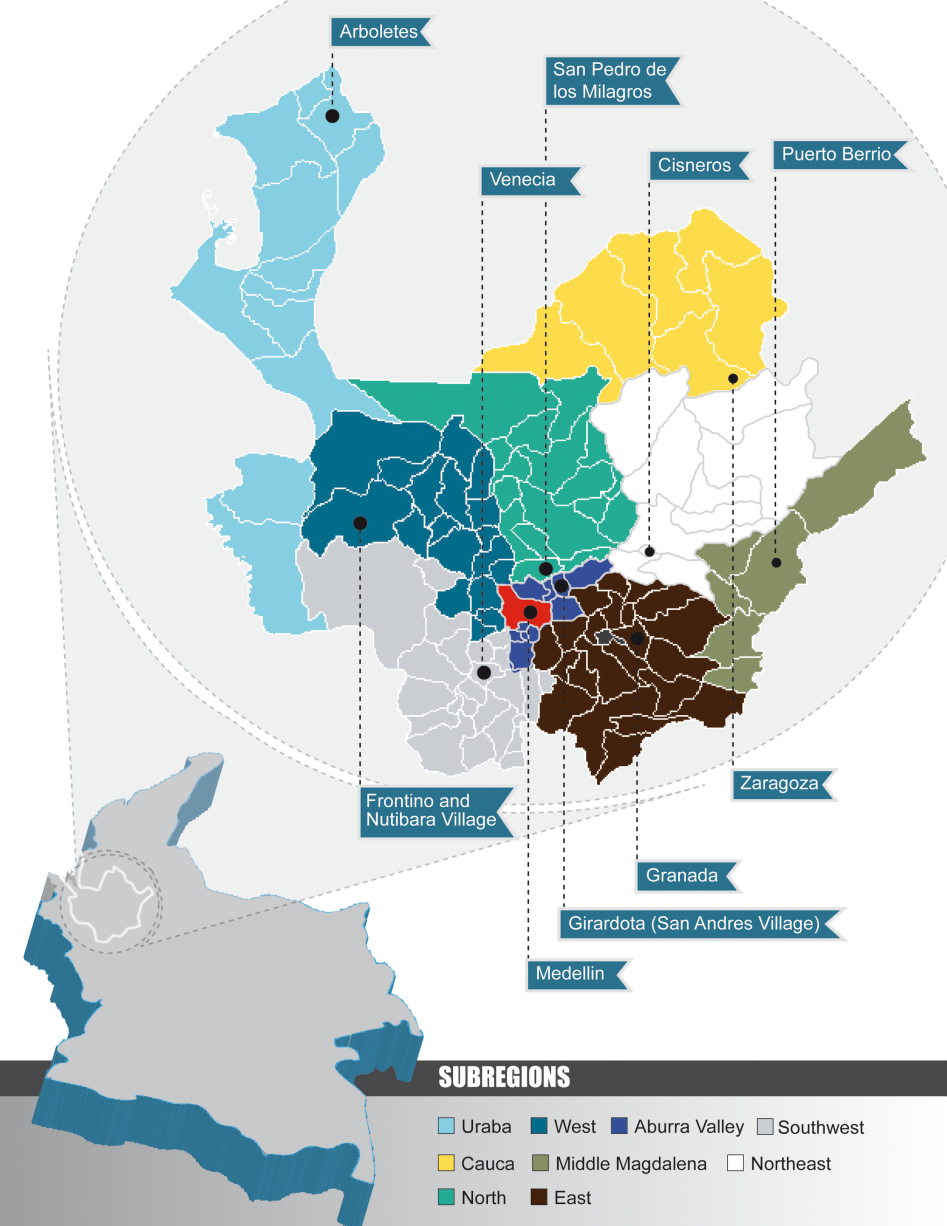
Geographic location of Antioquia state and the municipalities/villages included in the study.

Antioquia state is located in Northwest Colombia. Its capital, Medellin, is the second most important city of the country. The state is divided in nine administrative regions: Uraba, Cauca, North, Northeast, West, Southwest, East, Aburra Valley and Middle Magdalena (Fig 1). Antioquia is a state with high productivity in the agro-industrial scenario [36]; indeed, it is the most important state for commercial pig production farming in Colombia [37].

Considering that the main transmission route of HEV and Hepatitis A Virus (HAV) infection is fecal-oral and that HEV infection is currently not included in the guidelines of viral hepatitis in Colombia, the selection criteria for the municipalities was having the highest incidence of Hepatitis A in each one of the regions. Based on the data of the National System for Public Health Surveillance of Hepatitis A, eight municipalities and two villages were selected from the nine regions of Antioquia state for this study [38,39].

This project was approved by the ethics committee of the Faculty of Medicine of University of Antioquia and by the public health authorities of each one of the nine municipalities included. Moreover, approval was obtained from the water supply and wastewater treatment facilities operators of each municipality after socializing the project (Acueductos y Alcantarillados Sostenibles S.A. E.S.P. Arboletes, San Pedro de los Milagros, Venecia; Aguas del Puerto E.S.P. S.A. Puerto Berrio; E.S.P. Frontino, E.S.P. Granada, E.S.P. Cisneros; Junta local Nutibara, San Andrés).

### Water sample collection

Three samplings were carried approximately every four months in each municipality, between December 2012 and April 2014. The environmental samples were obtained without any treatment at drinking water plant (DWTP) or wastewater plant (WWTP). In seven municipalities with DWTP, the samples were obtained at the point of water entry at the plant; and in six municipalities and one village with WWTP or oxidation pond, the samples were obtained at the point of wastewater entry at the respective plant or pond (Table 1).

**Table 1:** Water treatment systems of the municipalities of Antioquia State included in the study

Region	Municipality/ Village	Population^+^	DWTP	WWTP
Uraba	Arboletes	31,039	Yes	Oxidation pond
Cauca	Zaragoza	25,173	Yes	No
West	Frontino	18,573	Yes	Oxidation pond
	Nutibara village	887	No	No
North	San Pedro de los Milagros	22,100	Yes	Yes
Aburra Valley	Girardota, San Andres village	42,818	No	Yes
Southwest	Venecia	13,352	Yes	Yes
East	Granada	9,436	Yes	Yes
Northeast	Cisneros	9,617	No	No
Middle Magdalena	Puerto Berrio	38,944	Yes	Oxidation pond

+ Data from the 2005 Census.

ND: No data available.

DWTP: Drinking water treatment plant, WWTP: wastewater treatment plant.

In one municipality and two villages without DWTP, water samples were obtained directly from the creek that supplies the local community; in all three cases there was only one water source for the community. Likewise, in municipalities without WWTP or oxidation pond, samples were obtained directly from the sewage outlet of the local community at a point in the sewage system of the village (Nutibara) or at the point of discharge to a creek (Zaragoza and Cisneros) (Table 1).

Five liters samples were collected at the main source of drinking water or at the creek and two liters sample of wastewater in each municipality/village. Samples were collected in plastic bottles and transported at 4°C to the laboratory for analysis.

### Viral concentration

Water samples obtained at the main source of drinking water or at the creek were concentrated by filtration and tangential ultrafiltration methodologies with 0.8 μm y 0.22 μm membranes, as previously described by Pelaez et al. [40]. The 5L sample volume was determined by the minimum volume to be processed by this technique [40].

**Fig 2:**
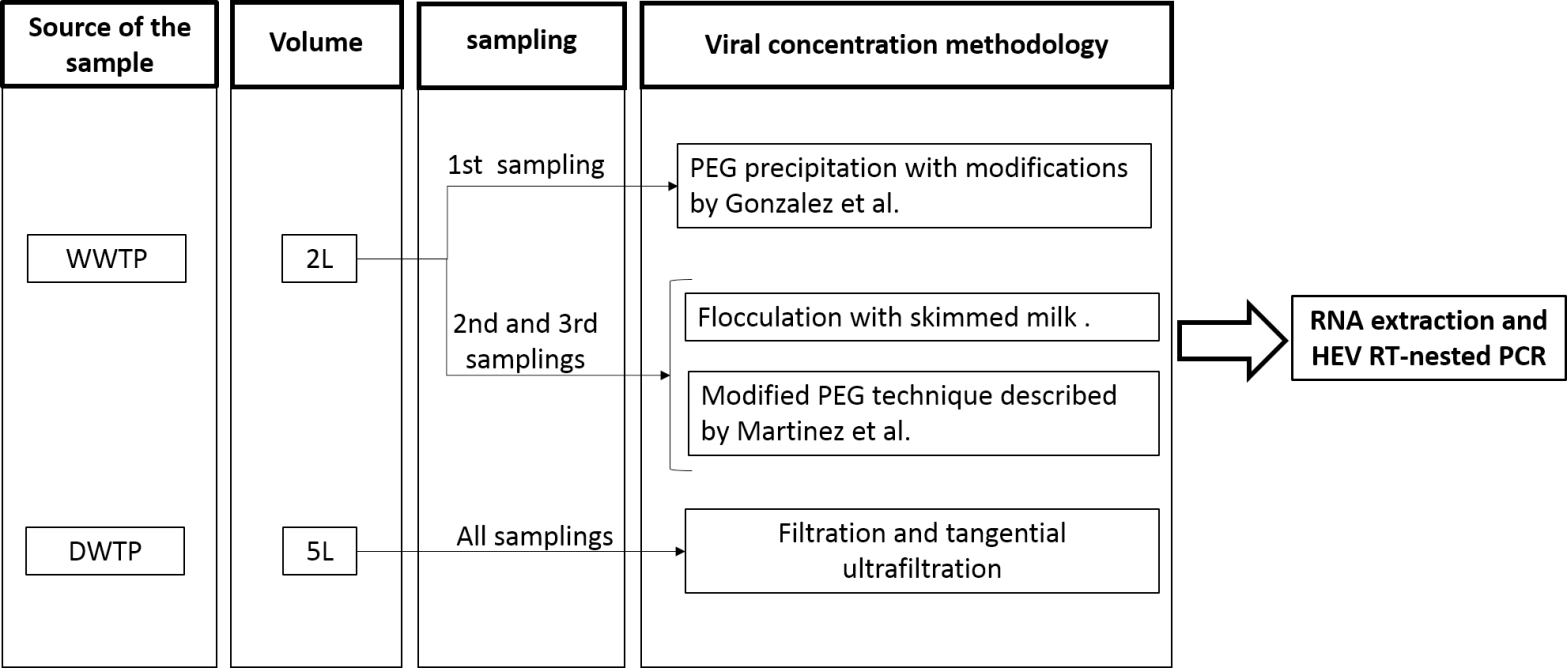
Sampling and viral concentration methodologies.

Wastewater samples from the first sampling were concentrated by polyethyleneglycol precipitation (PEG), following the protocol described by Sobsey [41] with modifications described by Gonzalez et al. [42]. Wastewater samples obtained in second and third samplings were divided in 50 mL and 1500 mL aliquots: the 50 mL aliquots were concentrated by flocculation with skimmed milk as described by Calgua et al. [43,44] and the 1500 mL aliquots were concentrated by modified PEG technique described by Martinez Wassaf et al. [11,45] (Fig 2). The viral concentrates were stored at—70°C. The volume of wastewater samples was determined by the minimum volume that the techniques required to work efficiently, and considering an adequate transportation and storage [42–44].

### Virus inoculation

Sewage samples positive for the viral genome by RT-PCR were inoculated on HepG2 cell cultures following the protocols described by Okamoto [46] and Tanaka et al. [47].

Briefly, cells were cultured in six-well culture plates and grown in Dulbecco’s Modified Eagle Medium (DMEM) supplemented with 10% FBS and 1% penicillin/streptomycin at 37 °C in a humidified 5% CO_2_ atmosphere.

Viral concentrates corresponding to sewage samples from Cisneros, San Pedro de los Milagros and Venecia were centrifuged at 4000 rpm for 10 minutes, and the supernatant was recovered and passed through a 0.22 μm filter. The filtrate was then diluted with PBS supplemented with 0.2% w/v bovine serum albumin (BSA), in a 1:1 or 1:5 ratio. Monolayers were washed three times with 1 mL of PBS and inoculated with 300 μL of the viral concentrate. Two hours after inoculation at 37°C, the inoculum was removed and 2 mL of DMEM supplemented with 2% FBS and 1% antibiotic were added. The culture was maintained at 37°C and 5% CO_2_. Twenty-four hours after, monolayers were washed 3–5 times with PBS and 2 mL of fresh growth medium were added. Cells were monitored daily for cytopathic effect (CPE) and 900 μL of medium were removed and stored at -70, and replaced with 1 mL of fresh growth medium. On day 30 post-inoculation, cells were harvested and frozen at—70°C for 72 hours, and then thawed at room temperature.

### RNA extraction

Viral RNA was obtained from the viral concentrates using a commercial kit (QIAamp Viral RNA Mini. QIAgen, Netherlands) following the manufacturer’s instructions.

RNA was obtained from supernatants and culture cells using the commercial kit described above or using TRIzol Reagent (Ambion by Life Technologies). Briefly, 175 μL of supernatant were mixed with 525 μL of TRIzol Reagent, then 100 μL of chloroform were added and then centrifuged at 12000 rpm for 15 minutes at 4°C. The aqueous phase was transferred to a new tube and 600 μL of isopropanol and 20 μL of glycogen 1 mg/mL were added. The sample was incubated for 60 minutes at -20°C, and then centrifuged at 12000 rpm for 45 minutes at 4°C. Ethanol 75% was added to the pellet, mixed and centrifuged at 12000 rpm for 45 minutes at 4°C. The pellet was dried at 55°C for 10 minutes and resuspended in 60 μL of nuclease-free water, then stored at -70^°^C.

### HEV RT—Nested PCR

A RT-nested PCR targeting the ORF2/3 region (nt 5258–5394) was performed to detect the HEV genome. Reverse transcription was performed in a final volume of 25 μL containing 5 μL of RNA and 200 U M-MLV (Invitrogen, USA), 1X First-strand buffer, 12 mM DTT, 800 μM dNTPs and 2 μM primer HE364 [48].

First and second round mixes contained 3 μL DNA, 1 U Biolase DNA Polymerase (Bioline, USA), 1X NH_4_ Reaction buffer, 2.2 mM MgCl_2_, 200 μM dNTPs, 0.5 μM of each primer, in a final volume of 25 μL. Primers HE361 (5’-GCRGTGGTTTCTGGGGTGAC-3’) and HE364 (5’-CTGGGMYTGGTCDCGCCAAG-3’) were used for the first round and primers HE366 (5’-GYTGATTCTCAGCCCTTCGC-3’) and HE363 (5’-GMYTGGTCDCGCCAAGHGGA-3’) for the second round. The cycling protocols were performed as described previously by Inoue et al. [48]. The final amplification product of 137 pb was visualized by 2% agarose gel electrophoresis stained with ethidium bromide. Each sample was analyzed in duplicate, with 5 μL of RNA and a 1:10 dilution of RNA as starting material. A supernatant from HEV-infected cell culture was gently donated by Dr. Shigeo Nagashima from the Jichi Medical University Tochigi-ken, Japan and was used as positive control.

### Phylogenetic analysis

Nucleotide sequences of amplicons were determined by automatic dideoxy-sequencing (Macrogen, Inc. Korea). Alignment and edition of the sequences were made on BioEdit 7.1.11 [49] and the phylogenetic analysis was performed on MEGA 5.2 [50]. The phylogenetic tree was constructed using the Neighbor-Joining method and Tamura 3-parameter + gamma distribution model, according to the Bayesian Information Criterion (BIC), with 1000 bootstrap replications. Pairwise distance test was performed to ensure no contamination between samples.

## Results

Sixty environmental samples (30 from the main source of drinking water plant or creek and 30 from sewage) were obtained from eight municipalities and two villages of Antioquia state between December 2012 and April 2014.

During the sampling, the rainfall conditions in each one of the municipalities were registered based on the information provided by the staff of the water supply and wastewater treatment facilities and by the Institute of Hydrology, Meteorology and Environmental Studies of Colombia (IDEAM). During the first sampling (December 2012 to May 2013), drizzle and isolated rains occurred, while throughout the second sampling (September to November 2013), strong and frequent precipitations took place during the visits to the municipalities. Finally, in the third sampling (December 2013 and April 2014), sporadic rains happened.

The HEV genome was detected in 23.3% (7/30) of the samples from the main source of drinking water plants, specifically from Granada, Zaragoza, Puerto Berrio, San Pedro de los Milagros, Frontino and from the creek that supplies the community of San Andres (Girardota). The HEV genome was detected in water samples obtained in two different samplings from Granada (Table 2).

**Table 2:** Detection of HEV genome on environmental samples throughout the three samplings in Antioquia State.

Sampling	Sample	Municipality/Village									
		San Andres Village*	San Pedro de los Milagros	Venecia	Granada	Cisneros	Arboletes	Zaragoza	Frontino	Nutibara Village*	Puerto Berrio
First sampling Dec 2012—May 2013	Source of DWTP	-	-	-	+	-	-	+	-	-	+
	Sewage	-	+	+	-	+	-	-	-	-	-
Second sampling Sept—Nov 2013	Source of DWTP	-	+	-	+	-	-	-	+	-	-
	Sewage	-	-	-	-	-	-	-	-	-	-
Third sampling Dec 2012—Apr 2014	Source of DWTP	+	-	-	-	-	-	-	-	-	-
	Sewage	-	+	-	-	-	-	+	-	-	-

DWTP: Drinking Water Treatment Plant

*Villages w/o DWTP; the water sample was obtained from the creek that supplies the community

**Fig 3:**
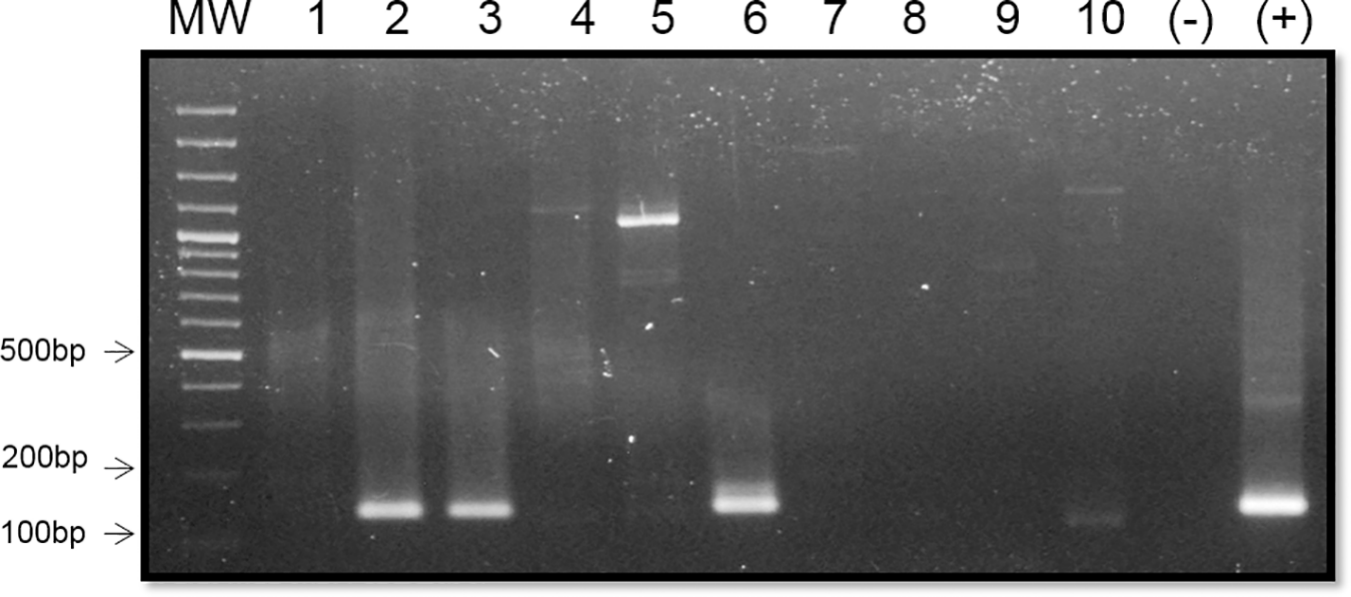
Detection of HEV genome on wastewater samples collected on first sampling. Representative results of the positive samples amplified by RT-nested PCR targeting the ORF2/3 region (137bp). MW: Molecular Weight. 1. Girardota village 2. San Pedro de Los Milagros 3. Venecia 4.Granada 5.Arboletes 6. Cisneros 7. Nutibara village 8. Frontino 9. Puerto Berrío 10. Zaragoza. (-) negative control (+) positive control. 2% agarose gel electrophoresis stained with ethidium bromide.

On wastewater samples, the HEV genome was detected with strong signal in 16.7% (5/30) of the samples, from San Pedro de los Milagros, Venecia, Cisneros and Zaragoza (Fig 3). Only in San Pedro de los Milagros two samplings were positive for the viral genome (Table 2).

Overall, environmental samples obtained from 80% of the municipalities and villages of Antioquia state tested positive for the HEV genome. In Zaragoza, Granada and San Pedro de los Milagros (30%), more than one sample tested positive for the HEV. For Granada, two samples from the source of drinking water plant were positive, while for Zaragoza and San Pedro de los Milagros, positive samples were detected in samples from the source of drinking water plant and wastewater. On the other hand, the HEV genome was not detected in any environmental samples from Arboletes and Nutibara village.

**Fig 4:**
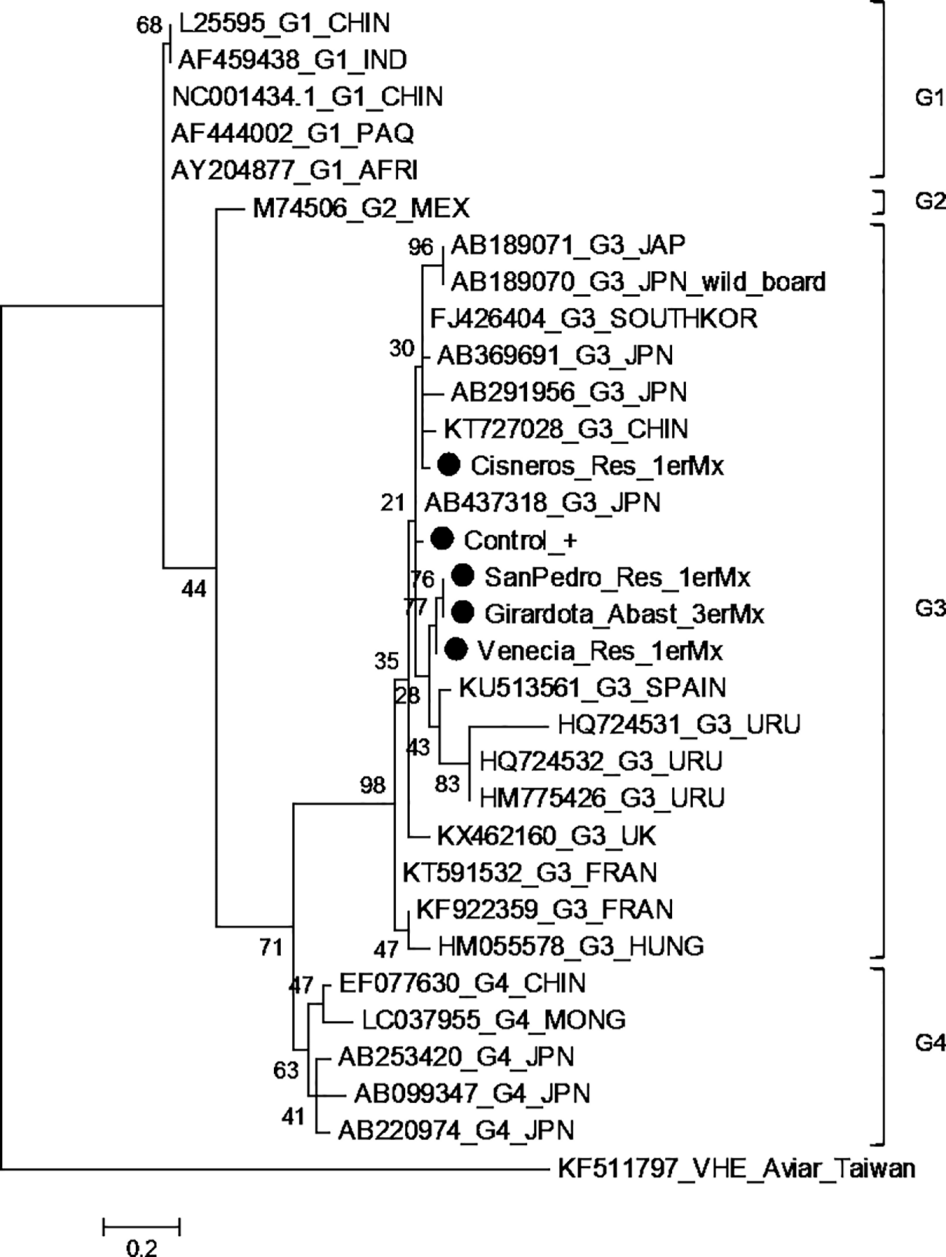
HEV phylogenic tree based on a fragment of 99 pb ORF2/3 HEV region. Based on the Bayesian Information Criterion (BIC), the model chosen for the phylogenetic analysis was Tamura 3-parameter + gamma distribution. The tree was constructed using the Neighbor-Joining method with 1000 bootstrap replicates. Samples obtained in this study are shown with a black circle (●). Clades for genotypes 1, 2, 3 and 4 are indicated with brackets. The GenBank accession number, genotype and country of isolation identify viral strains. Ven_Res_1erMx, SanPedro_Res_1erMx and Cisneros_Res_1erMx corresponded to wastewater sample collected on the first sampling at Venecia, San Pedro de los Milagros and Cisneros municipalities, respectively.

Three out of twelve positive PCR products were sequenced (Cisneros, Venecia, San Pedro de los Milagros), since 8/9 of the remaining samples showed weak PCR signal that may be related to low viral RNA concentration. Pairwise distance test showed no contamination between samples and the positive control. Phylogenetic analysis was performed using a database constructed with complete genome sequences of all four genotypes available in GenBank. After the edition process, a 99 bp sequence was used for the analysis. All samples clustered with genotype 3 sequences (Fig 4).

No CPE was observed in any of the cell cultures inoculated with sewage samples from San Pedro de los Milagros, Venecia or Cisneros. The supernatants recovered at day 1, 10, 20 and 30 were analyzed by RT-PCR and all were negative for the HEV genome. Lysed cells were also analyzed and tested negative for the viral genome.

## Discussion

This is the first report of HEV genome detection in environmental samples in Colombia. HEV was identified in at least one sampling in 7/8 municipalities and 1/2 villages of Antioquia state included in this study, and in 12/60 (20%) of all environmental samples analyzed. The HEV genome was detected in 23.3% (7/30) of the samples obtained at the main source of drinking water plants or at the creek and in 16.7% (5/30) of sewage samples (Table 2).

The number of positive samples from the first sampling (6/20), compared with the second (3/20) and third samplings (3/20) (Table 2), could be due to the differences on rainfall conditions between the samplings. As the first sampling period roughly coincided with the dry season, it is possible that the higher number of positive samples is related to the decrease of water flow during these months, while the second and third samplings coincided with the rainy season.

Indeed, the reduction in river flow levels during the summer resulting in lower dilution of viral particles and therefore, increased viral contamination when feces are discharged in the river was described in different regions of Europe and South America by Rusiñol et al. [51].

Furthermore, a seasonal pattern of HEV RNA positivity was described in sewage samples obtained during a period of two years in Northern India: the higher positivity rate for HEV RNA detected by PCR was found during the summer, while the lower positivity rate was found during the monsoon months [52]. A similar seasonal pattern was reported in a study carried out in Switzerland [53]. These studies provide evidence that changes in climatic conditions are related with the rate of detection of viral particles in environmental waters. Moreover, a dilution effect due to rain could be highly relevant in Colombia, considering that the sewage system is the same for wastewater and rainwater.

Although the rainfall pattern could explain the differences observed between the samplings in the present study, it is not possible to rule out the role of the viral concentration techniques used for sewage samples. In order to increase the possibility of detection, after the first sampling, two techniques for viral concentrations were used: PEG technique with some modifications, described by Martinez Wassaf et al. [11] and flocculation with skimmed milk technique [43,44]. The protocol used by Martinez Wassaf et al. differs from the protocol used for first sampling in two aspects: the pH control during the procedure and the ratio of weight sediment obtained from each sample and the volume of buffers [11]. On the other hand, Calgua et al. demonstrated that flocculation with skimmed milk technique was efficient and convenient in terms of its cost-effectiveness and sample processing time [44].

No significant differences were observed between these two techniques after the analysis of 20 sewage samples from the second and third samplings. However, the lack of a gold standard technique, the number of samples analyzed and the different environmental conditions between sampling do not allow the statistical analysis to estimate the efficiency of each technique. Nevertheless, the advantages of the flocculation technique described by Calgua et al. were confirmed in this study: sample volume, processing time and reagents cost [44].

The detection of the HEV genome in sewage from San Pedro de los Milagros, Cisneros, Venecia and Zaragoza suggests that the virus is circulating in these communities [11]. Interestingly, environmental samples obtained at San Pedro de los Milagros municipality were positive in all three samplings and from both the source of drinking water plant and wastewater, showing a continuous circulation of HEV in this municipality. This finding could be related to the porcine production in San Pedro de los Milagros, one of the most important in commercial pig production farming in Antioquia state, and even in the country. The results of the molecular detection of the HEV, as well as the conditions observed in the creek source of the DWTP that supplies this community, evidence a poor management of water and a deficient environmental and sewage disposal, increasing the risk of HEV transmission for its inhabitants [54,55].

The village of San Andres, that belongs to Girardota Municipality, lacks a DWTP. The creek that supplies this population tested positive for the viral genome on the third sampling, suggesting a risk of HEV infection to the local community.

The HEV genome has been also identified in environmental samples of several locations around the world. HEV has been detected in in river and sewage samples in Argentina [11], in river sample of Cambodia [12] and in samples of rivers that receive the wastewater from the residents nearby in the Philippines [13]. In The Netherlands, the HEV genome was found in samples from a river used for recreational purposes and drinking water, thus constituting a potential source of infection [14]; in Italy, HEV was detected in one raw sewage sample and in one river sample [15], while in Scotland, the viral genome was found in sewage [56]. In all of these reports, HEV genotype 3 was identified. Other countries that have reported HEV detection in urban sewage and wastewater include Spain, France, Switzerland, Norway, Tunisia, India and the USA [52,53,57–60].

Few studies have demonstrated the infectivity of HEV particles found in environmental samples. A study indicated that rhesus monkeys inoculated intravenously with viral concentrate positive by RT-PCR for HEV obtained from sewage became infected and excreted the virus in feces [57], but this approach is not readily available and it is expensive, since it requires non-standard laboratory animals. Furthermore, the absence of efficient cell-culture systems for the propagation of HEV has hinder its characterization [61], as well as the determination of the infectivity and survival of HEV present in environmental samples [62]. PLC/PRF/5 and A549 cell lines have been proved to be efficient for cell-culture adapted HEV strains [47,63].

We inoculated HepG2 cells with a viral solution prepared from sewage samples positive for HEV by RT- PCR, in order to determine if infectious viral particles were present in these samples. However, the HEV genome was not detected during the 30 days follow-up. The infectivity of the viral particles could not be rule out, considering that an inoculum with at least 2x10^4^ viral copies per well is necessary to detect the HEV RNA in culture supernatants, as described by Takahashi et al [64]. In addition, although it has been shown that HepG2 cells support the replication of cell-cultured adapted HEV strains [61], it can be less efficient for propagating wild type strains like the ones obtained in this study.

The phylogenetic analysis demonstrated that the strains isolated from the water samples belong to HEV genotype 3. Only two sequences from Latin America (Uruguay) were included in the analysis, since the region of other HEV sequences accessible on GenBank is different from the viral region analyzed in this study.

Genotype 3 is distributed worldwide and is described as zoonotic, being the swine its primary host; in addition, this genotype is associated with sporadic acute viral hepatitis [65]. This is in agreement with the epidemiological pattern that has been described in Latin America, where serological and molecular evidence of HEV infection with genotype 3 has been found in both human and swine samples throughout the region [21,22,27,66–69]. However, further studies are necessary to demonstrate the zoonotic transmission of HEV in countries of South America.

In Colombia, Pelaez et al. and Rendon et al. reported for the first time serological and molecular markers of HEV infection in patients diagnosed with acute viral hepatitis, respectively. Moreover, the strains of HEV obtained from these patients were identified as genotype 3 [29,30]. Betancur et al. reported a frequency of 11.25% for IgG anti-HEV in serum samples from pig farms workers in Antioquia [31], while Gutiérrez et al. detected the HEV genome in 26% of swine feces [33] and in 41.3% and 25% pig livers from slaughterhouses and grocery stores, respectively [35]. Forero et al. also identified the HEV genome in 26.9% of porcine stool samples from slaughterhouses of different subregions of Antioquia, which belonged to genotype 3 [34,70].

Since Hepatitis E infection is currently not included in the guidelines for the diagnosis and management of viral hepatitis in Colombia and therefore, it is not included in the National Surveillance Routine Program, we do not have information to determine whether our findings are consistent with acute hepatitis E cases occurring in the communities included in the study during the sampling period.

The presence of the HEV in source of drinking water plants samples and creeks should alert the public health system and the local health authorities. Importantly, easy and inexpensive measures can be taken in order to decrease the risk of infection for the communities. For water samples, chlorine treatment inactivates HEV and is an effective strategy to control the virus’ waterborne transmission: in order to achieve a 1-log HEV reduction (90% inactivation), a chlorine concentration between 0.15–0.12 mg/L x min is needed [20]. It is proposed that single-stranded RNA viruses, such as HEV, are particularly sensitive to UV radiation, and this was confirmed by an HEV inactivation of 99.99% achieved using low UV ranging between 195 and 269 J/m^2^; this means that current UV radiation guidelines (400 J/m^2^) represent a proper disinfection treatment for HEV [71]. Heating at temperatures higher than 70°C also appears to inactivate the virus [62].

The results of this study should draw the attention from local authorities of the municipalities without DWTP and/or WWTP, as these infrastructures are necessary for access to potable water and are relevant in terms of health and waterborne disease prevention [51]. Likewise, the zoonotic nature of HEV should concern both the swine industry and small farmers, so that appropriate management of waste and the quality of the meat for human consumption is guaranteed.

The findings of this study contribute to the knowledge and epidemiology of HEV in Colombia and in Latin America, and propose new challenges for the care and recovery of watersheds, given the possible fecal contamination from human and animal origin of the water supply. Waterborne transmission of HEV is a public health problem that must encourage the inclusion of HEV in the national protocols on public health surveillance as a pathogen of clinical relevance.
